# The impact of seasonal variations on IVF pregnancy outcomes: a retrospective cohort study in Jinan, China

**DOI:** 10.3389/fmed.2025.1681770

**Published:** 2025-10-21

**Authors:** Xin Hu, Heng-bing Li, Jing-yan Song, Hai-ning Yuan, Ying Xu, Kai-liang Ai, Zhen-gao Sun, Zhen-ni Mu

**Affiliations:** ^1^The First Clinical Medical College, Shandong University of Traditional Chinese Medicine, Jinan, China; ^2^Department of Reproduction and Genetics, The Affiliated Hospital of Shandong University of Traditional Chinese Medicine, Jinan, China

**Keywords:** *in vitro* fertilization, fresh embryo transfer, seasonal variation, meteorological factors, clinical outcomes, China

## Abstract

**Background:**

To evaluate the association between seasonal temperature variations and clinical outcomes of *in vitro* fertilization (IVF), aiming to provide theoretical foundations for optimizing protocol timing in reproductive medicine.

**Methods:**

This retrospective cohort study analyzed 2,551 first fresh IVF-embryo transfer (IVF-ET) cycles performed at a tertiary reproductive center of The First Affiliated Hospital of Shandong University of Traditional Chinese Medicine between January 2009 and January 2024. The study population comprised normo-ovulatory women aged < 35 years without uterine anomalies or severe male factor infertility (sperm concentration >1 × 106/mL). Cycles were stratified into four seasonal cohorts based on gonadotropin initiation dates: spring (March-May, *n* = 709), summer (June-August, *n* = 787), autumn (September-November, *n* = 640), and winter (December-February, *n* = 415). Primary outcomes included clinical pregnancy rate (CPR), live birth rate (LBR), miscarriage rate (MR), and Full-Term delivery rate (FTBR), analyzed through multivariable logistic regression models adjusting for mean daily temperature (°C), relative humidity (%), and daylight hours (h).

**Results:**

Compared with the winter control group, risk of miscarriage in cycles initiated in spring showed a statistically significant increase (95% CI 1.019, 2.846; *P* = 0.042). Although CPR showed no seasonal variation (spring: 54.30%, summer: 52.22%, autumn: 50.47%, winter: 50.36%; *P* = 0.464), the spring cohort exhibited a numerically higher Full-Term delivery rate (39.07 vs. 34.22%; *P* = 0.105). Sensitivity analysis using weighted analysis to balance sample sizes across groups revealed significantly higher full-term birth rates in spring compared to winter (*P* = 0.046) and the live birth rate in spring was also significantly higher than in winter (*P* = 0.029). For each unit increase in sunlight intensity on the trigger day, the probability of successful pregnancy decreases to approximately 0.978 times the original value (OR = 0.978 per lux-unit increase, 95% CI 0.960–0.997; *P* = 0.025).

**Conclusion:**

Seasonal microenvironmental factors during ovarian stimulation may modulate IVF success trajectories, suggesting potential benefits of climate-adaptive protocol personalization in temperate monsoon regions.

**Clinical trial registration:**

This is a retrospective case-control study.

## Background

Seasonal variations in human reproduction have been widely observed and studied. Although these differences are not as pronounced in humans as in other mammals, research has indeed shown that a range of hormone levels fluctuate with the seasons. Researchers have noted that most pituitary hormones peak in late summer, while the effector hormones produced by downstream organs reach their zenith in winter and spring. These seasonal hormonal fluctuations may be linked to seasonal trends in human reproduction (1). The study by Klaudia et al. found that extreme heat can not only negatively impact hormonal balance but may also more directly affect oocyte quality and disrupt mitochondrial function, thereby influencing maternal transcription in the embryo (2). A clinical study analyzing birth records of 56 million newborns across 445 different geographical units in the United States provided macroscopic evidence of the impact of high temperatures on pregnancy. Research has shown that when ambient temperatures reach or exceed 32.2°C, birth rates are ~0.97 times higher than during periods when temperatures range from 15.6 to 21.1°C. Notably, birth rates decrease within the following 2 days, indicating that elevated temperatures may contribute to an increased risk of preterm birth. However, 2 days later, the birth rate decreased, suggesting that hot weather may increase the likelihood of preterm delivery (3). This biphasic response suggests activation of prostaglandin-mediated thermoregulatory pathways, which may precipitate preterm labor. Furthermore, fluctuations in basal body temperature (BBT), a crucial indicator of female reproductive health, exhibit seasonal patterns that are highly significant for understanding the interaction between the menstrual cycle and environmental factors. A study observing seasonal variations in BBT found that temperatures during both the follicular and luteal phases were higher in summer and lower in winter (4). This finding laid the foundation for further exploring how seasons influence pregnancy outcomes by affecting the menstrual cycle. On the other hand, seasonal changes in light exposure impact melatonin secretion. Melatonin not only plays an indispensable role in the hormonal regulation of the female reproductive axis (5, 6) but also regulates endometrial morphology and embryo implantation, and maintains pregnancy by modulating hormone levels (7, 8). Data on term birth rates from natural pregnancies show seasonal variation (9–12), which has sparked interest in whether *in vitro* fertilization (IVF) cycles are similarly influenced by season.

IVF involves pharmacological ovarian stimulation to induce multiple follicular development, followed by oocyte retrieval and laboratory fertilization with sperm. This process enables subsequent embryo culture and developmental potential assessment prior to uterine transfer for pregnancy establishment. Given the demonstrated sensitivity of IVF outcomes to environmental factors—particularly seasonal variations—a thorough investigation of this relationship is warranted. A Henan Province (China) cohort study revealed significant seasonal influences on IVF clinical pregnancy rates, with autumn and winter yielding higher proportions of high-quality embryos and improved pregnancy outcomes compared to spring and summer (11). Conversely, in Tanzania's tropical savanna climate, peak conception rates occurred during the hottest months rather than the rainy season (13), suggesting region-dependent fertility seasonality. In studies evaluating meteorological parameters, some have attributed seasonal effects on IVF outcomes to changes in temperature (14). Some research suggests that the regularity of seasonal changes in food supply, temperature, humidity, and atmospheric pressure is not comparable to that of photoperiod (15). However, research on this topic has yielded inconsistent results thus far, with some studies identifying significant seasonal or temperature effects, while others have found none (16). Discrepancies in results may stem from variations in study populations and their geographical environments, as well as inconsistencies in the criteria used across studies to assign patients to specific seasons (e.g., based on the date of stimulation, oocyte retrieval, or embryo transfer). Therefore, further research is needed to understand the impact of climate and seasonal temperatures on IVF success rates in different regions.

Located in Shandong Province, China, Jinan exhibits a temperate continental monsoon climate with marked seasonal variations in temperature and precipitation: summers (June–August) are hot-humid with concentrated rainfall, while winters (December–February) feature cold-dry conditions accompanied by frequent temperature inversions. At our tertiary reproductive medical center, we analyzed a retrospective cohort of over 2,000 completed IVF cycles to investigate how seasonal variations during ovarian stimulation initiation influence embryo developmental competence and pregnancy outcomes. To better understand and apply these seasonal factors, we utilized multivariate logistic regression analysis to comprehensively consider potential influencing factors on *in vitro* fertilization outcomes. This type of research provides valuable directions for clinical practice, helping us optimize treatment strategies and improve IVF success rates. The relationship between these seasonal factors and IVF success rates still requires further clarification through additional research to achieve the ultimate goal of maximizing human reproduction.

## Materials and methods

### Study design

We conducted a retrospective cohort study of women who underwent IVF-ET at the Reproductive and Genetic Center of the Affiliated Hospital of Shandong University of Traditional Chinese Medicine between August 1, 2009, and January 1, 2024. The included patients underwent treatment with various ovarian stimulation protocols. A total of 2,551 women completed their first fresh embryo transfer cycle and met the inclusion and exclusion criteria. All patients underwent conventional IVF fertilization without ICSI. Jinan, Shandong, has a temperate monsoon climate with distinct seasons, characterized by warm and rainy summers, cool and dry autumns, cold winters with little snow, and clear seasonal variation throughout the year. Based on the date of ovarian stimulation initiation to the date of oocyte retrieval, all cycles were categorized into spring (March to May), summer (June to August), autumn (September to November), and winter (December to February). If the duration from the start of ovarian stimulation to oocyte retrieval spanned two different seasons, the cycle was classified according to the season with the longer proportion of days.

### Inclusion and exclusion criteria

Eligible participants were required to meet all of the following criteria:

(1) Reproductive-aged women (20 ≤ age < 35 years);
(2) Completed fresh embryo transfer in the index stimulation cycle.

Exclusion criteria encompassed:

(1) Hepatic/renal impairment (serum ALT > 2 × ULN, eGFR < 60 mL/min/1.73 m^2^) or cycle discontinuation due to non-medical factors;
(2) ≥2 prior pregnancy losses (intrauterine demise beyond 20 gestational weeks);
(3) Anatomic/endocrine pathologies including but not limited to: endometriosis (ASRM stage III-IV), adenomyosis (JZ thickness > 12 mm), congenital uterine anomalies (ESHRE/ESGE classification), endometrial polyps >10 mm, intramural fibroids distorting the cavity, previous uterine surgery with residual myometrium <3 mm, pelvic tuberculosis (GeneXpert-confirmed), cervical length < 25 mm, hydrosalpinx with fluid accumulation >3 cm3, PCOS (Rotterdam criteria), recurrent implantation failure (≥3 euploid embryo transfers), recurrent miscarriage (≥3 consecutive losses), or thyroid dysfunction (TSH > 4.0 mIU/L);
(4) Parental chromosomal abnormalities (karyotype analysis);
(5) Severe male factor infertility (sperm concentration < 1 × 106/mL and total motility < 5% and normal morphology < 1% by strict Kruger criteria).

### Meteorological data

Meteorological data for Jinan City, Shandong Province, covering the period from 1 January 2009 to 30 March 2024, was downloaded from the China Meteorological Data Network (http://data.cma.cn/). The data includes daily mean temperature, daily mean humidity, and duration of sunshine. First, meteorological exposure time windows are defined by selecting key time points or phases within each IVF cycle, such as the trigger day (in IVF treatment, the “trigger day” refers to the day when specific medication is administered to induce final egg maturation and ovulation after follicles have developed to an appropriate stage), the egg retrieval day, or the phase from the gonadotropin day (Gn day) to the egg retrieval day. Calculate the average temperature, average humidity, and average duration of daylight exposure for each specific time window, subsequently conducting a logistic regression analysis to assess their association with pregnancy outcomes.

### Controlled ovarian hyperstimulation protocol

**Figure 1:**
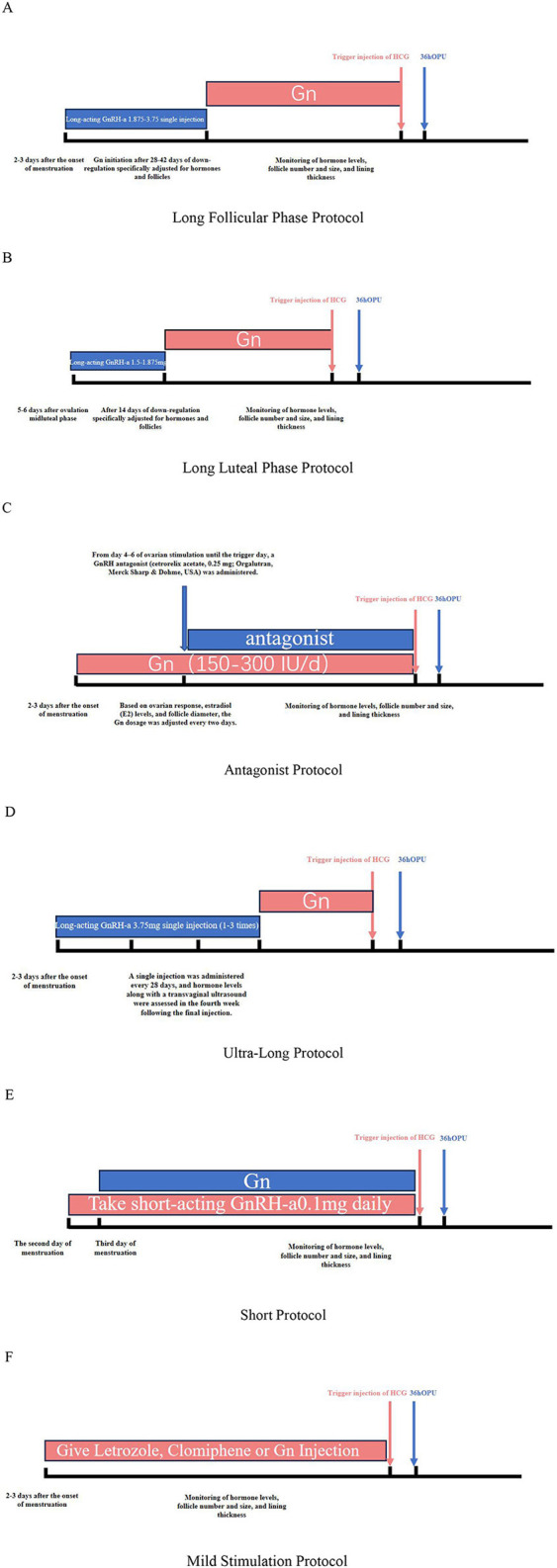
**(A)** Long follicular phase protocol. **(B)** Long luteal phase protocol. **(C)** Antagonist protocol. **(D)** Ultra-long protocol. **(E)** Short protocol. **(F)** Mild stimulation protocol.

Schematic diagrams outlining the standardized procedures for the commonly employed ovarian stimulation protocols (Long Follicular Phase Protocol, Long Luteal Phase Protocol, Antagonist Protocol, Ultra-Long Protocol, and Mild Stimulation Protocol) are presented in Figure 1. Using the antagonist protocol as an example, all patients underwent a flexible GnRH antagonist regimen. Ovarian stimulation was initiated on day 2 or 3 of the menstrual cycle with gonadotropin administration (Gn, Gonal-F). The initial dose (typically 150–300 IU/day) was determined by physicians according to patient age, antral follicle count (AFC), baseline FSH, E_2_ levels, and BMI. Dosage adjustments were made every 2–3 days based on ovarian response, serum E_2_ levels, and ultrasound-monitored follicular development. GnRH antagonist (Cetrotide) was introduced when the leading follicle reached 14–15 mm in diameter or exceeded 12 mm with serum estradiol >200 pg/mL. Final triggering was performed using 250 μg Ovitrelle when either: (1) three leading follicles exceeded 17 mm, or (2) two dominant follicles achieved an average diameter of 18 mm. Transvaginal ultrasound-guided oocyte retrieval followed 24–36 hours post-trigger.

### Fresh embryo transfer protocol

Fresh embryo transfer eligibility required concurrent fulfillment of strict biochemical and ultrasonographic criteria on the day of human chorionic gonadotropin (hCG) administration: endometrial thickness 8–15 mm, serum progesterone < 1.5 ng/mL, serum estradiol < 5000 pg/mL, and oocyte yield < 15, regardless of preexisting hydrosalpinx or endometrial polyps status. Standardized luteal support was initiated immediately post-retrieval, comprising either intramuscular progesterone (40 mg/day) or vaginal progesterone gel (Crinone^®^ 8%, 90 mg/day). Cleavage-stage embryo transfer was conducted on day 3 post-retrieval for patients without evidence of (1) post-procedural complications (defined as febrile events or hemorrhagic requiring intervention) or (2) early ovarian hyperstimulation syndrome (manifesting as ascites volume >100 mL and/or hematocrit >48%). Luteal supplementation was maintained throughout the post-transfer period until definitive pregnancy assessment.

### Pregnancy outcome measures

Primary endpoints included clinical pregnancy rate (CPR) and live birth rate (LBR). Secondary outcomes comprised term delivery rate (TDR) and clinical miscarriage rate (CMR). Clinical pregnancy was strictly defined as ultrasonographic confirmation of ≥1 intrauterine gestational sac with cardiac activity at 6–8 gestational weeks. Live birth required delivery of any live fetus demonstrating postnatal vital signs (spontaneous respiration, heartbeat, or voluntary muscle movement) for ≥1 minute, irrespective of gestational duration (≥22 weeks), placental status, or plurality. Term pregnancy was operationally defined as delivery at ≥37°/7 gestational weeks. Early miscarriage denoted spontaneous pregnancy loss prior to 126/7 gestational weeks, excluding biochemical pregnancies and ectopic gestations. Calculation methods for embryo quality indicators: 2PN fertilization rate (2PN fertilization count/egg retrieval count × 100) 2PN cleavage rate (2PN cleavage count/2PN fertilization count × 100) High-quality embryo rate (high-quality embryo count/2PN cleavage count × 100).

### Statistical analysis

dATA processing and statistical analyses were conducted using IBM SPSS Statistics v25.0 (IBM Corp., Armonk, NY). Continuous variables demonstrating non-normal distribution (assessed via Shapiro-Wilk test) were summarized as median (interquartile range, IQR), with skewed data dispersion explicitly characterized by 25th−75th percentile ranges. Missing values were imputed through multivariate random forest algorithms (missForest package v1.5), with imputation consistency validated using Cronbach's α coefficient > 0.80. Intergroup comparisons employed: ANOVA with Bonferroni *post-hoc* adjustment for Gaussian-distributed variables, Kruskal-Wallis test for nonparametric data, and χ^2^/Fisher's exact tests (applied when expected cell counts < 5) for categorical variables. Multivariable modeling incorporated: linear regression [β coefficients with 95% confidence intervals (CI)] for continuous endpoints and adjusted logistic regression [odds ratios (OR) with 95% CI] for binary outcomes. Statistical significance was defined as two-tailed α = 0.05 without multiplicity correction, consistent with exploratory secondary analysis design.

## Results

### Overall Similarity in Patient Demographic Characteristics

A total of 2,551 fresh embryo transfer cycles from eligible women were analyzed following rigorous inclusion/exclusion criteria. Cycles were stratified into four seasonal cohorts based on the interval between gonadotropin initiation and oocyte retrieval: spring (*n* = 709), summer (*n* = 787), autumn (*n* = 640), and winter (*n* = 415). To ensure statistical robustness, the ten protocols were regrouped into three major categories based on the core criterion of “whether pituitary suppression was employed and its duration.” Classic long downregulation group (GnRH-a ≥ 14 days): Long protocol, long-acting long protocol, follicular phase long protocol, luteal phase short-acting long protocol, Long-acting long-phase luteal phase protocol, ultra-long protocol; Short downregulation/trigger group: Short protocol, ultra-short protocol, antagonist protocol; No downregulation/minimal stimulation group (pituitary function not suppressed): Micro-stimulation protocol, natural cycle/modified natural cycle.

Demographic comparisons demonstrated homogeneous distributions across seasons for age, BMI, pre-retrieval follicular count, basal hormonal profiles (FSH, LH, E_2_, P), and infertility etiologies (all *P* > 0.05). Notably, significant seasonal disparities were observed in infertility duration (*P* = 0.005), underscoring the necessity of multivariable adjustments in subsequent analyses (Table 1).

**Table 1:** Comparison of baseline and clinical characteristics across different seasons.

**Variable**	**Spring (*n* = 709)**	**Summer (*n* = 787)**	**Autumn (*n* = 640)**	**Winter (*n* = 415)**	***P***
Age (years)	31 (28, 33)	31 (29, 34)	32 (29, 33)	31 (29, 33)	0.893
Duration of infertility (years)	3 (2, 4)	3 (2, 4)	3 (2, 4)	3 (1, 4)	0.005
BMI (kg/m^2^)	22.90 (20.50, 26.20)	22.55 (20.63, 25.38)	22.60 (20.70, 25.80)	23.30 (20.70, 26.20)	0.104
Antral follicle count	9 (6, 12)	9 (6, 12)	9 (6, 11)	9 (6, 12)	0.413
Basal FSH^a^ (IU/L)	7.21 (6.08, 8.43)	7.24 (6.25, 8.54)	7.09 (6.10, 8.33)	7.18 (5.96, 8.42)	0.278
Basal LH^a^ (IU/L)	4.53 (3.15, 6.02)	4.67 (3.26, 6.01)	4.64 (3.30, 5.90)	4.60 (3.34, 6.18)	0.787
Basal E2^a^ (pg/mL)	50.86 (31.12, 119.25)	49.28 (32.60, 102.76)	46.50 (30.80, 85.55)	53.00 (34.35, 110.70)	0.082
Basal *P*^a^ (ng/mL)	0.68 (0.40, 1.50)	0.69 (0.30, 1.30)	0.64 (0.37, 1.30)	0.755 (0.37, 1.54)	0.116
Non-pregnant (*n*)	320	356	294	186	0.985
Primary Infertility (*n*)	389	431	346	229	0.366
long GnRH agonist protocol (*n*)	390	386	309	211	0.052
Short GnRH agonist protocol (*n*)	313	394	324	195	
Minimal stimulation protocol (*n*)	6	7	7	9	

^a^Missing values were imputed using the random forest algorithm.

### Seasonal variations influence embryo numbers and fertilization rates

No significant seasonal variations were observed in gonadotropin (Gn) duration, total Gn dosage, oocyte yield, trigger-day (The term “Trigger-day” as used in the document, refers to the day of hCG administration) endometrial thickness, or embryos transferred. IVF normal fertilization rates exhibited significant seasonal fluctuation (*P* = 0.020), peaking in winter (62.0%) vs. spring (57.0%). Day 3 embryo counts differed markedly (*P* = 0.008), with autumn demonstrating the highest yield (median 5) compared to other seasons (median 4). Clinical pregnancy rates ranged from 50.4% (winter) to 54.3% (spring) (*P* = 0.464), while miscarriage rates varied between 10.1% (winter) and 15.3% (spring) (*P* = 0.126). Live birth rates approached statistical significance (*P* = 0.054), with spring achieving the highest rate (51.6%) vs. autumn (42.5%) (Table 2).

**Table 2:** Embryo quality indicators and pregnancy outcomes by season.

**Variable**	**Spring (*n* = 709)**	**Summer (*n* = 787)**	**Autumn (*n* = 640)**	**Winter (*n* = 415)**	***P***
Duration of Gn use (days)	10 (9, 12)	10 (8, 12.5)	10 (8, 12)	10 (9, 12)	0.166
Total Gn dose (IU)	2,300 (1,693.75, 3,000)	2,175 (17,25, 2,850)	2,250 (1,800, 3,000)	2,250 (1,725, 2,850)	0.488
Number of oocytes retrieved	9 (7.00, 12.00)	9 (6, 12)	9 (6, 12)	9 (6, 12)	0.111
Endometrial thickness on trigger day (mm)	11 (9.30, 12.70)	11 (9.3, 12.5)	11 (9.2, 12.6)	11 (9.0, 12.6)	0.862
Number of embryos transferred	2 (2, 2)	2 (2, 2)	2 (2, 2)	2 (2, 2)	0.899
IVF normal fertilization rate	0.57 (0.44, 0.75)	0.60 (0.49, 0.78)	0.60 (0.45, 0.76)	0.62 (0.48, 0.78)	0.02
Number of good-quality embryos	2 (0, 2)	2 (0, 2)	2 (0, 2)	2 (0, 2)	0.829
Number of 2PN^a^	4 (4, 7)	4 (4, 7)	4 (4, 7)	4 (4, 7)	0.488
Number of 2PN cleavage	4 (3, 6)	4 (3, 6)	4 (3, 6)	4 (3, 6)	0.344
2PN cleavage rate (%)	100.00 (82.42, 100.00)	100.00 (83.33, 100.00)	100.00 (80.00, 100.00)	100.00 (81.68, 100.00)	0.891
High-quality embryo rate (%)	20.00 (0.00, 33.33)	20.00 (0.00, 33.33)	23.61 (0.00, 42.26)	16.67 (0.00, 33.33)	0.07
Number of D3 embryos	4 (2, 7)	4 (2, 7)	5 (2, 8)	4 (2, 7)	0.008
Clinical pregnancy rate (%)	385 (54.30%)	411 (52.20%)	323 (50.47%)	209 (50.36%)	0.464
Miscarriage rate (%)	59 (15.32%)	62 (12.07%)	39 (12.06%)	21 (10.05%)	0.126
Live birth rate (%)	277 (51.62%)	253 (43.96%)	208 (42.50%)	142 (46.99%)	0.054
Full-Term delivery rate (%)	277 (39.07%)	253 (32.15%)	208 (32.50%)	142 (34.22%)	0.105

^a^2PN, 2 pronuclei.

### Spring associates with a significantly elevated risk of miscarriage compared to winter

Compared to winter reference, spring demonstrated a modest elevation in clinical pregnancy rates (OR 1.171, 95% CI 0.919–1.493; *P* = 0.202) and term birth rates (OR 1.233, 95% CI 0.957–1.587; *P* = 0.105), though both lacked statistical significance. Live birth rates showed no seasonal disparity (*P* > 0.50). It is noteworthy that compared with winter, the risk of miscarriage increased by 70.3% in spring (OR 1.703, 95% CI 1.019, 2.846; *P* = 0.042). In contrast, fluctuations in all outcomes during summer and autumn did not reach statistical significance (Table 3).

**Table 3:** Association between seasons and reproductive outcomes.

**Season**	**Clinical pregnancy rate**			**Full-term delivery rate**			**Live birth rate**			**Miscarriage rate**		
	***P***	**OR**	**95% CI**	***P***	**OR**	**95% CI**	***P***	**OR**	**95% CI**	***P***	**OR**	**95% CI**
Spring	0.202	1.171	0.919, 1.493	0.105	1.233	0.957, 1.587	0.076	1.254	0.976, 1.612	0.042	1.703	1.019, 2.846
Summer	0.539	1.077	0.849, 1.367	0.468	0.911	0.708, 1.172	0.834	0.974	0.759, 1.249	0.069	1.604	0.964, 2.671
Autumn	0.973	1.004	0.784, 1.286	0.563	0.926	0.713, 1.203	0.807	0.968	0.748, 1.254	0.48	1.217	0.706, 2.101
Winter		1			1			1			1	
Intercept	0.022	1.015		0	0.051		0	0.549		0	0.053	

*OR*, odds ratio; *CI*, confidence interval. The reference season is winter. *P* trend is calculated by treating winter as an ordinal variable.

### Increased solar exposure on trigger day is associated with reduced probability of clinical pregnancy

To evaluate stage-specific environmental effects on assisted reproductive technology (ART) outcomes, stratified analyses were performed according to critical treatment milestones: (1) gonadotropin (Gn) stimulation phase, (2) oocyte retrieval day, and (3) trigger day. Conduct independent analysis of meteorological parameters such as ambient temperature, relative humidity, and duration of sunshine for each time window (Table 4).

**Table 4:** Comparison of meteorological data across different annual periods.

**Temperature**	**2009–2012**	**2013–2016**	**2017–2020**	**2021–2024**	***P***
Spring	15.33 ± 7.34	16.51 ± 6.68	16.72 ± 6.77	16.12 ± 6.18	0.147
Summer	26.52 ± 2.97	26.59 ± 2.81	26.90 ± 2.86	26.85 ± 2.66	0.087
Autumn	15.14 ± 6.64	15.62 ± 7.05	15.85 ± 6.51	16.36 ± 6.82	0.206
Winter	1.58 ± 4.32	1.86 ± 3.94	1.98 ± 4.13	1.94 ± 4.72	0.087
**Humidity**	**2009–2012**	**2013–2016**	**2017–2020**	**2021–2024**	***P***
Spring	45.08 ± 17.49	45.46 ± 17.78	45.59 ± 16.60	46.62 ± 18.86	0.955
Summer	65.42 ± 18.17	66.59 ± 14.22	64.86 ± 16.49	64.38 ± 16.51	0.481
Autumn	58.69 ± 18.54	58.62 ± 18.24	56.98 ± 16.66	58.19 ± 17.50	0.544
Winter	49.05 ± 18.77	51.35 ± 31.03	50.48 ± 19.00	51.28 ± 21.53	0.536
**Duration of sunshine**	**2009–2012**	**2013–2016**	**2017–2020**	**2021–2024**	***P***
Spring	7.37 ± 4.06	7.68 ± 3.90	7.78 ± 3.88	7.54 ± 4.47	0.433
Summer	6.48 ± 4.30	6.45 ± 4.08	6.45 ± 4.38	6.88 ± 4.39	0.379
Autumn	5.77 ± 3.69	6.16 ± 3.82	6.04 ± 3.77	6.17 ± 3.97	0.117
Winter	5.44 ± 2.71	5.53 ± 3.26	5.30 ± 3.43	5.49 ± 3.63	0.079

Notably, trigger-day solar exposure demonstrated a dose-dependent inverse association with pregnancy success in unadjusted models (OR 0.978 per 100 W/m^2^ increase, 95% CI 0.960–0.997; *P* = 0.025). Each incremental unit of sunlight intensity during trigger administration corresponded to a 2.2% reduction in clinical pregnancy probability, establishing statistically significant effect modification by this temporal exposure (Table 5).

**Table 5:** Association between meteorological factors and reproductive outcomes.

**Meteorological factor**	**Clinical pregnancy rate**			**Full-term delivery rate**			**Live birth rate**			**Miscarriage rate**		
	***P***	**OR**	**95% CI**	***P***	**OR**	**95% CI**	***P***	**OR**	**95% CI**	***P***	**OR**	**95% CI**
Mean temperature^a^	0.864	1.001	0.992, 1.009	0.402	0.996	0.988, 1.005	0.976	1	0.991, 1.009	0.287	1.009	0.992, 1.026
Mean humidity^a^	0.94	1	0.994, 1.006	0.212	0.996	0.990, 1.002	0.954	1	1.000, 1.000	0.301	0.994	0.982, 1.006
Duration of sunshine^a^	0.16	0.972	0.935, 1.011	0.903	1.003	0.962, 1.045	0.256	0.977	0.938, 1.017	0.685	1.016	0.942, 1.096
Retrieval temperature	0.969	1	0.993, 1.006	0.41	0.997	0.989, 1.005	0.091	1.008	0.992, 1.025	0.157	1.011	0.996, 1.027
Retrieval humidity	0.648	1.001	0.977, 1.015	0.871	1	0.995, 1.004	0.452	1.002	0.992, 1.006	0.325	0.995	0.989, 1.004
Retrieval duration of sunshine	0.698	0.996	0.977, 1.015	0.585	0.994	0.974, 1.014	0.482	1	0.993, 1.007	0.321	1.019	0.982, 1.058
Trigger day temperature	0.777	1.001	0.993, 1.009	0.709	0.998	0.990, 1.007	0.729	1.001	0.993, 1.010	0.422	1	0.991, 1.022
Trigger day humidity	0.177	1.003	0.994, 1.009	0.541	0.999	0.994, 1.003	0.864	1	0.996, 1.005	0.425	1.003	0.995, 1.011
Trigger day duration of sunshine	0.025	0.978	0.960, 0.997	0.775	0.997	0.977, 1.017	0.464	0.993	0.973, 1.012	0.397	0.984	0.949, 1.021

Meteorological factors include values measured at embryo retrieval and transfer days.

^a^Average temperature, humidity and duration of sunshine refer to the mean values of meteorological data recorded between the commencement date of ovulation induction and the date of oocyte retrieval.

### Weighted analysis confirms significantly higher live birth and full-term delivery rates in spring

Due to the smaller sample size in winter, regression analysis was conducted after balancing sample sizes across groups using weighted analysis. The association between seasonal parameters and clinical pregnancy differed slightly from results described in our overall analysis. Weighted analysis revealed that the spring season was associated with a higher rate of full-term delivery (*P* = 0.046, OR = 1.233, 95% CI = 1.004, 1.514) and live birth rate (*P* = 0.029, OR = 1.254, 95% CI = 1.023, 1.538) were significantly higher than in winter. The miscarriage rate in spring increased by 70.3% compared to winter, even before weighting (*P* = 0.010) (Table 6).

**Table 6:** Association between weighted seasons and reproductive outcomes.

**Season**	**Clinical pregnancy rate**			**Full-term delivery rate**			**Live birth rate**			**Miscarriage rate**		
	***P***	**OR**	**95% CI**	***P***	**OR**	**95% CI**	***P***	**OR**	**95% CI**	***P***	**OR**	**95% CI**
Spring	0.118	1.171	0.961, 1.428	0.046	1.233	1.004, 1.514	0.029	1.254	1.023, 1.538	0.01	1.703	1.134, 2.558
Summer	0.46	1.077	0.884, 1.313	0.383	0.911	0.738, 1.124	0.801	0.974	0.792, 1.198	0.832	1.604	1.064, 2.420
Autumn	0.966	1.004	0.824 1.224	0.47	0.926	0.751, 1.142	0.76	0.968	0.787, 1.191	0.79	1.217	0.790, 1.876
Winter		1			1			1			1	
Intercept	0.839	1.015		0	0.52		0	0.549		0	0.053	

### Subgroup analyses reveal pronounced seasonal effects in classic long protocols

In the subgroup analysis restricted to the short downregulation/flare group (GnRH-a administered for days 1–6 only, utilizing the flare-up effect), the association between seasonal parameters and clinical outcomes was broadly consistent with the findings described in our overall analysis. Compared with winter, spring demonstrated a 35.3% higher live birth rate (*P* = 0.049, OR = 1.353, 95% CI = 1.001, 1.827), whilst clinical pregnancy rates decreased with increasing trigger day temperature (*P* = 0.020, OR = 0.971, 95% CI = 0.946, 0.995). The miscarriage rate significantly increased in spring (*P* = 0.012, OR = 2.167, 95% CI = 1.182, 3.970) (Table 7).

**Table 7:** Scheme I: association between seasonal and meteorological factors and pregnancy outcomes.

**Season**	**Clinical pregnancy rate**			**Full-term delivery rate**			**Live birth rate**			**Miscarriage rate**		
	***P***	**OR**	**95% CI**	***P***	**OR**	**95% CI**	***P***	**OR**	**95% CI**	***P***	**OR**	**95% CI**
Spring	0.016	1.397	1.063, 1.836	0.244	1.192	0.892, 1.567	0.286	1.163	0.881, 1.536	0.25	1.382	0.796, 2.398
Summer	0.007	1.478	1.115, 1.958	0.902	1.019	0.760, 1.365	0.52	1.099	0.825, 1.464	0.005	2.134	1.260, 3.613
Autumn	0.041	1.342	1.012, 1.778	0.473	1.113	0.831, 1.490	0.236	1.189	0.893, 1.584	0.785	1.086	0.599, 1.970
Winter		1			1			1			1	
Intercept	0.507	0.936		0	0.54		0	0.611		0	0.06	
Sunlight duration for trigger	0.447	0.99	0.966, 1.015	0.672	0.97	1.970, 1.020	0.337	0.988	0.964, 1.013	0.877	0.952	0.952, 1.043

### Among patients with good ovarian function undergoing the classic long downregulation protocol

(GnRH-a continuous use ≥14 days, downregulation followed by stimulation, in patients with good ovarian function), this protocol is suitable for patients with good ovarian function whose reproductive systems are more likely to respond positively to environmental influences. The prolonged treatment cycle exposes patients to seasonal environmental factors for a longer duration, amplifying the cumulative effects of environmental factors. Consequently, the association between seasonal parameters and clinical pregnancy rates is more pronounced (Table 8).

**Table 8:** Scheme II: association between seasonal and meteorological factors and pregnancy outcomes.

**Season**	**Clinical pregnancy rate**			**Full-term delivery rate**			**Live birth rate**			**Miscarriage rate**		
	***P***	**OR**	**95% CI**	***P***	**OR**	**95% CI**	***P***	**OR**	**95% CI**	***P***	**OR**	**95% CI**
Spring	0.777	0.959	0.719, 1.290	0.098	1.289	0.954, 1.740	0.049	1.353	1.001, 1.827	0.012	2.167	1.182, 3.970
Summer	0.103	0.792	0.599, 1.048	0.182	0.814	0.602, 1.101	0.338	0.863	0.638, 1.167	0.958	1.018	0.517, 2.005
Autumn	0.054	0.76	0.575, 1.005	0.091	0.77	0.569, 1.101	0.122	0.787	0.581, 1.066	0.305	1.393	0.739, 2.626
Winter		1			1			1			1	
Intercept	0.335	1.103		0	0.5		0	0.489		0	0.046	
Sunlight duration for trigger	0.02	0.971	0.946, 0.995	0.552	1.008	0.981, 1.036	0.462	1.01	0.983, 1.038	0.532	0.983	0.933, 1.036

Owing to the insufficient sample size of Scheme Three (*n* = 29), which fails to meet the fundamental requirements for statistical inference, this study excluded it from subsequent analyses to avoid introducing bias in the results.

### After multivariate adjustment, the correlation between seasonal factors and *in vitro* fertilization outcomes weakened

Prior to constructing the multivariate models, we performed univariate screening to identify potential predictors of the four pregnancy outcomes. After adjusting for key confounders—including age, basal progesterone (*P*), trigger-day endometrial thickness, total high-quality embryos in IVF, number of 2PN zygotes on day 1 (IVFD1_2PN), and number of day 3 embryos transferred (D3)—no statistically significant seasonal variation (spring, summer, or autumn vs. winter) was observed in live birth rates (all *P* > 0.05).

Basal FSH, basal E2, basal progesterone, trigger day endometrial thickness, and IVF normal fertilization rate adjusted for miscarriage rate. When controlling for other variables, the comparativ effects of spring, summer, autumn, and winter on miscarriage rates were not significant.

For clinical pregnancy rates, adjustments were made for age, Gn duration, infertility duration, follicle count, trigger-day endometrial thickness, total high-quality IVF embryos, and total fresh embryos transferred. Results indicate no statistically significant differences in clinical pregnancy rates between different seasons (spring, summer or autumn versus winter)(all *P* values > 0.05). Similarly, term delivery rates were adjusted for age, Gn duration, endometrial thickness, total high-quality IVF embryos, fresh embryos transferred, and D3 embryos transferred. After accounting for these variables, spring showed a marginally beneficial trend in term delivery rates compared to winter, though this did not reach conventional statistical significance.

After balancing sample sizes across groups in the weighted analysis, age, baseline progesterone levels, endometrial thickness on trigger day, total number of high-quality IVF embryos, IVF D1_2PN, number of embryos transferred on day 3, number of 2PN fertilized, number of 2PN cleaved, 2PN cleavage rate, and high-quality embryo rate were adjusted for live birth rate. After controlling for other variables, the spring live birth rate was higher than that in winter (*P* = 0.006, OR = 1.355, 95% CI = 1.093, 1.680), consistent with the overall analysis. After adjusting for basal *P*, trigger day endometrial thickness, total number of high-quality IVF embryos, number of 2PN-stage embryos, 2PN cleavage rate, high-quality embryo rate, number of 2PN-stage fertilized oocytes, number of embryos transferred on day 3, baseline FSH, baseline E2, and IVF normal fertilization rate, the results remained similar to the univariate analysis, with spring miscarriage rates higher than winter (*P* = 0.009, OR = 1.750, 95% CI = 1.150, 2.665); Gn days, duration of infertility, number of oocytes retrieved, total number of high-quality IVF embryos, total number of fresh embryos transferred, number of 2PN fertilized oocytes, and medication protocol, consistent with previous findings, revealed no significant difference between spring, summer, autumn, and winter (*P* < 0.05). Trigger day endometrial thickness, 2PN cleavage rate, quality embryo rate, number of cleavages, D3 transfer number, basal FSH, basal E2, IVF normal fertilization rate, age, Gn days, duration of infertility, number of oocytes retrieved, total number of high-quality IVF embryos, total number of fresh embryos transferred, number of 2PN fertilized oocytes, and medication regimen. This analysis was consistent with the overall findings, showing a significant increase in full-term delivery rates in spring compared to winter (*P* = 0.021, OR = 1.298, 95% CI = 1.040, 1.622).

## Discussion

The seasonal patterns observed in human birth rates demonstrate the significant influence of environmental factors and climatic conditions on reproductive physiology. These variables affect not only timing and the success of natural conception (17, 18) but may also influence ART outcomes, particularly those of IVF (10, 19). Our statistical analysis suggests that seasonal variations and ambient temperature may impact embryo development and clinical pregnancy rates during ovarian stimulation cycles that precede oocyte retrieval. This observation aligns with recent research: Leslie et al. reported a positive correlation between temperature and both IVF implantation and clinical pregnancy rates (9), while other studies have indicated that warmer seasons (summer and autumn) are associated with higher conception rates compared to winter (20). Notably, research conducted during transitions to and from daylight saving time documented a 45% reduction in live birth rates following embryo transfer (21).

The scientific literature confirms that environmental temperature affects ovarian function and follicular development. Experimental evidence suggests that heat stress may cause oxidative damage to ovarian tissue in mice (22), potentially impairing reproductive capacity. These findings indicate that the ovary is sensitive to thermal changes, with elevated temperatures triggering granulosa cell apoptosis, altering steroid hormone secretion, and modifying the expression of related genes. Simultaneously, subtle temperature variations may influence uterine receptivity through changes in endometrial thickness and quality, consequently affecting implantation success (23). Current research continues to investigate how temperature, daylight exposure, and humidity at the time of oocyte retrieval may collectively influence pregnancy outcomes.

Our analysis of embryo quality parameters revealed seasonal variations in both IVF fertilization rates and the number of day 3 (D3) embryos. These differences suggest that environmental factors may affect gamete quality or early embryonic development, particularly during the period between fertilization and cleavage the D3 stage. Notably, compared with winter cycles, IVF cycles commencing in spring showed a significantly increased miscarriage rate.

Jinan's spring climate (March-May) exhibits several distinctive features that may contribute to these outcomes: rapid temperature increases with substantial diurnal variation (of about 10–14°C, representing the largest annual fluctuation), dry and windy conditions with low levels of precipitation, and high evaporation rates. Both metabolic chamber studies and ecological research indicate that diurnal temperature variation significantly drives individual energy metabolism (24). For pregnant women, the inherent upregulation of basal metabolic rate and circulatory load during gestation heightens sensitivity to environmental temperatures (25). Such thermal fluctuations markedly increase miscarriage risk through mechanisms including elevated white blood cell counts and subsequent disruption of the immune microenvironment (26). The natural characteristics of spring, including the renewal of vegetation and environmental revitalization, may also exert positive effects on mental health, potentially reducing anxiety and depression associated with pregnancy complications (27). This effectively establishes a stringent natural selection barrier at the onset of pregnancy, eliminating fragile embryos unable to withstand such stress. Those embryos that successfully navigate this phase may possess greater vitality, thereby achieving higher developmental success rates under the subsequent favorable conditions of spring—enhanced light exposure, temperature, and nutrition—ultimately translating into increased live birth and full-term delivery rates. These combined environmental factors enhance IVF success rates by regulating physiological rhythms (spring provides optimal conditions for endometrial synchronization and receptivity compared to gradually increasing daylight hours). Once stabilized, melatonin not only exhibits potent antioxidant effects but also promotes placental angiogenesis, ensuring sustained fetal nourishment (28). This study reveals a seemingly paradoxical phenomenon: compared to winter, spring exhibits a higher rate of early miscarriage, yet ultimately yields significantly higher rates of live birth and full-term delivery. This indicates that seasonal factors influence pregnancy outcomes not through a linear, singular mechanism, but rather via a complex, phased process.

The observed inverse relationship between trigger-day daylight duration and clinical pregnancy rates suggests a potential role for circadian rhythm disruption. Light exposure regulates circadian rhythms primarily through its effects on the suprachiasmatic nucleus (SCN), where it influences cellular oscillator organization and suppresses melatonin secretion (29, 30). As a key circadian regulator, melatonin maintains and coordinates biological rhythms (31, 32), and disturbances have been linked to ovulatory dysfunction, reduced fertility, increased miscarriage rates, and abnormal fetal development (33). These effects occur through multiple mechanisms, including hormonal regulation, cell cycle control, gene expression modulation, and apoptosis (34).

The SCN regulates reproductive function through several neuroendocrine pathways. Two major SCN neuropeptide cell populations produce vasoactive intestinal peptide (VIP) and arginine vasopressin (AVP), which participate in luteinizing hormone (LH) surge generation and ovulation (35). Additional research indicates that SCN-derived AVPergic projections target kisspeptin neurons in the anteroventral periventricular nucleus (AVPV), which subsequently stimulate gonadotropin-releasing hormone (GnRH) neurons in the preoptic area to mediate estradiol-induced LH surges (36). These findings demonstrate that the SCN coordinates GnRH system activity through both direct and indirect pathways, ensuring properly timed ovulation and supporting pregnancy maintenance.

Circadian regulation also extends to prolactin, a hormone critical for pregnancy maintenance. VIPergic SCN neurons innervate oxytocin neurons in the hypothalamic paraventricular and supraoptic nuclei, thereby influencing prolactin secretion (37) and pregnancy outcomes. Melatonin, a principal circadian output, enhances progesterone production by human granulosa and luteal cells (6), reduces estradiol levels, and increases endometrial glandular density (7), while simultaneously stimulating prolactin secretion and inhibiting oxytocin release (8), collectively promoting embryo implantation and pregnancy maintenance. Furthermore, melatonin facilitates trophoblast differentiation and human chorionic gonadotropin (hCG) secretion (38), supporting placental development and fetal growth. Clinical studies demonstrated that melatonin supplementation can improve hormonal profiles, oocyte quality, pregnancy rates, and reduce the incidence of miscarriage (39, 40).

Extended daylight exposure prolongs circadian cycle duration (41), while bright morning light enhances nocturnal sleep quality by regulating melatonin secretion (42). Experimental pinealectomy studies have shown that melatonin deficiency increases estradiol production, suppresses FSH and LH secretion through negative feedback, and inhibits ovulation (43, 44), while also causing endometrial hyperplasia and structural abnormalities incompatible with pregnancy maintenance (45). These mechanisms may explain our observation of a reduced likelihood of pregnancy with increasing trigger-day daylight duration.

Moreover, the vitamin D receptor is widely expressed in the ovaries, endometrium, and placenta (46). Increasing evidence indicates that vitamin D influences ovarian reserve function by directly regulating the transcription of steroid synthase expression and AMH production (47). Furthermore, vitamin D modulates the cell cycle, promotes granulosa cell proliferation, and enhances follicular development to improve oocyte quality (48). Mice lacking the vitamin D receptor exhibit impaired follicular development and abnormal hormone secretion (49). Studies using animal models investigating the role of the vitamin D receptor in fertility and reproductive function reveal that vitamin D deficiency reduces female fertility by inducing uterine developmental defects (50). During pregnancy, increased expression of the mitochondrial enzyme 1α-hydroxylase (encoded by CYP27B1) in the endometrium elevates serum vitamin D levels to meet heightened calcium demands (51). This supports embryonic development, reduces gestational complications, and maintains pregnancy. Under the influence of 1,25(OH)_2_D3, endometrial cells exhibit increased expression of osteopontin (a progesterone-regulated adhesion molecule mediating implantation and decidualisation), confirming vitamin D's role in facilitating embryo implantation (52). Human skin synthesizes vitamin D through exposure to ultraviolet B (UVB) radiation. For instance, studies indicate that the Earth's axial tilt results in a lower angle of solar radiation, reducing UVB intensity. Particularly in high-latitude regions, vitamin D synthesis is virtually impossible during winter (53), hence clinical pregnancy rates are slightly lower in winter compared to spring and summer. However, the relationship between UVB radiation and vitamin D synthesis is non-linear. Excessive UVB exposure may lead to saturation and degradation of vitamin D synthesis (54): after sufficient skin exposure to UVB, pre-vitamin D3 (7-dehydrocholesterol) reaches its upper limit. Excessive UVB may cause pre-vitamin D3 to convert into inactive photoproducts, failing to further increase serum vitamin D levels. Consequently, intense summer sunlight does not necessarily significantly elevate vitamin D levels; rather, it may slightly limit synthesis through photodegradation. Combined with heat stress from high temperatures, this contributes to lower rates of full-term delivery and live births in summer compared to spring. Furthermore, clinical pregnancy rates progressively decline as trigger sunlight intensity increases.

This study has several notable strengths. First, Jinan's temperate monsoon climate, characterized by marked seasonal temperature variations, provides an ideal setting for investigating thermal effects on IVF outcomes. The region's consistent monsoon patterns facilitate reliable seasonal classification and time-series analysis. Second, our approach of tracking environmental exposures throughout the entire ovarian stimulation phase (from cycle initiation to oocyte retrieval) allows for a more accurate assessment of cumulative effects on hormonal profiles, oocyte quality, and embryonic development. This method yields more biologically meaningful and individualized seasonal categorizations. Third, our inclusion of diverse ovarian stimulation protocols in this retrospective analysis enhances the statistical power and generalizability of our findings while minimizing selection bias.

Several limitations should be acknowledged. First, we could not fully account for potential seasonal variations in semen quality. Second, as with all retrospective studies, residual confounding from unmeasured variables may influence the observed associations between seasonal factors and reproductive outcomes. Third, confounding bias arising from the smaller sample size in winter seasons distorts the estimation of the true effect of seasonality on pregnancy outcomes.

Our findings suggest potential clinical applications for optimizing IVF protocols. If confirmed by larger prospective studies, these results could support season-specific timing of ovarian stimulation to maximize success rates and minimize miscarriage risk. This research provides preliminary evidence for an association between seasonal factors and ART outcomes, potentially informing more personalized treatment strategies while stimulating further investigation into the physiological mechanisms underlying these relationships.

## Conclusion

This study systematically evaluated seasonal variations in *in vitro* fertilization (IVF) procedures and outcomes across all four seasons. Key findings indicate a 70.3% increase in miscarriage rates during spring compared to winter (*p* < 0.05). Moreover, following sensitivity analysis with weighted balancing of sample sizes across groups, spring demonstrated significantly higher FDR and LBR than winter. However, summer and autumn outcomes—including CPR, FDR, LBR, and miscarriage rates—showed no significant deviation from the winter baseline. Notably, increased daylight hours on the trigger day showed a negative correlation with pregnancy success rates, whereas daylight hours during ovarian stimulation and egg retrieval did not significantly correlate with CPR.

Important limitations include the retrospective design and residual confounding from unmeasured variables. Future prospective trials incorporating granular environmental data (e.g., UV index, temperature fluctuations) and hormonal profiling are required to mechanistically clarify seasonality's role in reproductive outcomes.

## Data Availability

The raw data supporting the conclusions of this article will be made available by the authors, without undue reservation.
